
JOURNAL INFORMATION
==============================
NLM Title Abbreviation: Eur Psychiatry
Journal ID: Eur Psychiatry
NLM Journal ID: 9111820
Journal ID: EPA

European Psychiatry

ISSN: 0924-9338
EISSN: 1778-3585
Publisher: Cambridge University Press

ARTICLE INFORMATION
==============================
PMCID: PMC9412709
DOI: 10.1192/j.eurpsy.2020.11
Article ID: S0924933820000115
Article version: 1
Subjects: Abstracts

State of the Art

Publication date: 2020 Jul
Electronic publication date: 2020 Sep 4
Volume: 63
Issue: Suppl 1
Issue title: Abstracts of the 28th European Congress of Psychiatry
First page: S618
Last page: S619
Copyright: © The Author(s) 2020
Copyright year: 2020
Copyright holder: The Author(s)
License: This is an Open Access article, distributed under the terms of the Creative Commons Attribution licence (http://creativecommons.org/licenses/by/4.0/), which permits unrestricted re-use, distribution, and reproduction in any medium, provided the original work is properly cited.
License URL: https://creativecommons.org/licenses/by/4.0/

Page count: 2

==============================

Article ID: SOA002
Subjects: Abstract, Bipolar Disorder: Keys to Successful Outcomes

Bipolar disorder: keys to successful outcomes

Vieta E.
University of Barcelona , Department of Psychiatry and Psychology, Barcelona , Spain

Article ID: SOA003
Subjects: Abstract, Eating Disorders: From Neurobiological Factors Involved to Therapy

Eating disorders: from neurobiological factors involved to therapy

Treasure J.
King’s College, Institute of Psychiatry , London , United Kingdom

